# Extensive Removable Bridge-Work

**Published:** 1889-11

**Authors:** J. Marion Edmunds

**Affiliations:** New York City


### EXTENSIVE REMOVABLE BRIDGE-WORK.[1]

BY J. MARION EDMUNDS, D.D.S., NEW YORK CITY.

There is not a branch of any profession so exacting in its requirements upon the skill, taste, and judgment of the operator as dental prosthesis. Ranking, as prosthetic dentistry does, with the higher branches of fine art, we need not be surprised, when we reflect how little it is valued by the average dentist, that there are so many failures and so many pieces of unsightly work; for mechanical dentists are many, and dental artists few. Our casual observations on the street, beach, in the drawing-room, and at our office in everyday practice is all that is necessary to verify these statements. Here these disgraceful productions of the dental mechanic are only too frequently seen in the mouths of the confiding unfortunates. With the advantages of the present at his command, it is not only possible for the dental artist to replace missing teeth with such perfection as to deceive the closest observer, but also to restore all the lost symmetry of the face, and thereby to bring back the youthful expression, long since passed away.

The development of the past fifty years is truly marvellous. Fifty years ago dental prosthesis had just advanced from a curved ivory denture to a swaged gold plate with porcelain facings in place of the human and ivory teeth. A few years later, after the invention of rubber, and when competition among the dental mechanics became greater for something cheap, rubber was adopted as a base for artificial teeth, and it produced even cheaper dentures than had been anticipated by the most sanguine charlatan. But, thanks to the few brave ones who held the standard of our

---

[1] Read before the New Jersey State Dental Society, at its nineteenth annual session at Asbury Park, July 17, 1889.

profession high and ever worked for advancement, with each renewed effort some great result was achieved. When Dr. Bing, of Paris, conceived the idea of attaching one missing tooth to two remaining teeth, by soldering a piece of gold wire to the back of an artificial facing, and building it with gold foil into cavities, either natural or artificial, then the genius of the American dentist was called upon to improve and advance this idea to a height so sublime that the original is lost in obscurity. Drs. Webb, Beers, Low, Starr, Evans, and Brown have all contributed much to advance this new era in denistry,—Dr. Webb improving on Dr. Bing's method, Dr. Beers inventing the gold cap, Dr. Low soldering one or more facings to these gold caps and forming what is known as the Low bridge. Dr. R. Walter Starr, of Philadelphia, to my mind made a decided improvement on the Low bridge when he invented the removable bridge, which had many advantages over all other kinds of bridge-work. My object has been, since I commenced this new, beautiful, and most useful method of prosthesis, to extend its sphere of usefulness as far as possible, and to-day I have the pleasure to present to this society an original method which has given my patients and myself the most gratifying results.

As æsthetic prosthesis is the standard "*par excellence*," all factors in the process must work to this end. The size and color of the teeth should correspond with the age and temperament of the patient; the material used as a base in the construction should be as near the color of the natural tissue as possible.

As the soft tissues and membrane play an important part in the support of this process, they must be examined with care; and, before putting on a cap or inserting a bridge, any deviation from the normal should be treated with remedial agents until health is fully restored. The next step is to make another cap to telescope over the one already in place. Then take an impression of the tooth-space, and of a few of the teeth in front of the space that is to be filled. From this impression run a model, and cast a duplicate of zinc in sand; then run a counter-die and swage a narrow rim of gold plate to rest on the gum as a saddle. This must now be placed in position on the model, the outer cap waxed in place and soldered there. This accomplished, it may be placed in the mouth, the bite taken, the teeth waxed in position and articulated. The model should be scraped so that the saddle may fit firmly upon the gum. This is done to allow for the expansion of the plaster, otherwise there might be an injurious strain on the tooth supporting the bridge. If from absorption there should be a deficiency or falling in of the buccal walls, the natural expression can be restored by carrying the gold plate well up on the buccal walls of the alveolar ridge, adding wax where the deficiency has occurred.

The advantages of having merely a gold rim held firmly in contact with the gums, covering only the alveolar ridge, held there by the laws of adhesion and mechanical force, and removable at the will of the patient, are obvious. Counter-sunk teeth are invariably the best substitute, resembling as they do the natural organs, and affording the patient a more perfect enunciation and articulation than can be obtained in any other way. I have inserted two full upper dentures which are giving perfect satisfaction. My first case was that of a lady who had been wearing for ten years a full upper plate, with the exception of two superior third molars. The alveolar ridge around these was almost completely destroyed. I could pass a probe from the margin of the gum to at least three-fourths the length of the root. The teeth were so loose that it seemed absurd to think of saving them. The tissue of the entire arch was also badly inflamed. This plate did not fit the arch or articulate with a single tooth. The lower teeth, eight of which were in the anterior part of the inferior arch, were allowed to occlude on the plate three-eighths of an inch posterior to the corresponding superior teeth. There was a strip of gold vulcanized on the plate to prevent the inferior teeth wearing through the rubber. You can well imagine what a severe expression the shortening of the bite gave, adding to this the extension of the upper teeth and lips in the manner previously stated. Without describing this artificial deformity further, I will only say I have seen few worse caused by freaks of nature. After considerable persuasion on the part of the patient to save the two remaining molars, and insert a bridge if possible, I decided to try. Removing the tartar and *débris* thoroughly, I used aromatic sulphuric acid, half-strength, on pledgets of cotton, packing them as close to the walls of the socket as I could, and allowing them to remain for two hours at a time. This treatment was continued for a week. Then, after scarifying the tissue with a small burr, treatment was continued for one week longer, with injections of peroxide of hydrogen and iodide of zinc. By this time healthy granulations had appeared and the teeth were firmly set. I then prepared the teeth and capped them, and in one more week inserted full bridge constructed as previously described. This case has been in the mouth thirteen months, the patient wearing it with great comfort, whilst the remaining teeth are nearly firm, notwithstanding they support a full denture.

